# Spectrum of genetic variants in 306 patients with non-syndromic hearing loss from Croatia

**DOI:** 10.3325/cmj.2024.65.198

**Published:** 2024-06

**Authors:** Ivona Sansović, Ana-Maria Meašić, Adriana Bobinec, Leona Morožin Pohovski, Ljubica Odak, Katarina Vulin, Bernarda Lozić, Mijana Kero, Sanda Huljev Frković, Silvija Pušeljić

**Affiliations:** 1 Department of Medical and Laboratory Genetics, Endocrinology and Diabetology, Children’s Hospital Zagreb, University of Zagreb School of Medicine, Zagreb, Croatia; 2 University of Applied Health Sciences, Zagreb, Croatia; 3 Scientific Centre of Excellence for Reproductive and Regenerative Medicine, University of Zagreb School of Medicine, Zagreb, Croatia; 4 Paediatric Diseases Department, University Hospital of Split, Split, Croatia; 5 University of Split School of Medicine, Split, Croatia; 6 Department of Pediatrics, University Hospital Centre Zagreb, University of Zagreb, School of Medicine, Zagreb, Croatia; 7 Department of Pediatrics, University Hospital Center Osijek, Osijek, Croatia

## Abstract

**Aim:**

To determine the spectrum and frequency of disease-causing variants in patients with non-syndromic hearing loss (NSHL) and to investigate the diagnostic yield of the applied genetic methods.

**Methods:**

The study enrolled 306 unrelated patients with childhood-onset, mild-to-profound NSHL referred to Children’s Hospital Zagreb for genetic testing between March 2006 and October 2023. The *GJB2* variants were analyzed with the multiplex ligation-dependent probe amplification method and Sanger sequencing of the coding region of the *GJB2* gene. In 21 patients negative for *GJB2* biallelic variants, clinical exome sequencing (CES) was performed.

**Results:**

Among 234 disease-associated *GJB2* alleles detected, 19 were clinically relevant, of which 18 were reported as pathogenic/likely pathogenic. The c.35delG variant accounted for 73.5% of the mutated alleles. More than half of the patients with biallelic *GJB2* variants (64/110, 58.2%) were 35delG homozygotes. Seventeen non-GJB2 variants were found in 10 genes (*TECTA*, *NOG*, *SLC26A4*, *PCDH15*, *TMPRSS3*, *USH2A*, *GATA3*, *MYO15A*, *SOX10*, *COL2A1*) in 11 participants, and 5 variants (in *TECTA*, *NOG*, *PCDH15*, and *SOX10*) were novel (29.4%).

**Conclusion:**

We were able to elucidate the genetic cause of hearing loss in 121 patients, with an overall diagnostic rate of 39.5%. The c.35delG was the most common variant. CES allowed us to diagnose almost half of the patients with HL; to distinguish NSHL from the syndromic form of HL in cases where the phenotype was unclear or where symptoms were absent from an early age; and to discover novel variants.

Genetic factors cause 60% of congenital hearing impairment, and about 70% of congenital hearing loss (HL) is non-syndromic (1). A major challenge in the study of congenital non-syndromic hearing loss (NSHL) is extreme genetic heterogeneity, whereby the same or very similar clinical phenotype is the result of pathogenic variants in a considerable number of genes. To date, 153 genes have been associated with NSHL, of which 63 are associated with autosomal dominant (AD) loci DFNA (DeaFness, Non-syndromic, A indicates autosomal dominant inheritance), 86 with autosomal recessive (AR) loci DFNB (DeaFness, Non-syndromic, B indicates autosomal recessive inheritance), seven are sex-linked genes, and nine are mitochondrial associated genes. Nevertheless, different mutations in one gene (for example, *GJB2, TECTA, MYO7A*) can cause recessive and dominant forms of NSHL, which makes genetic counseling for affected families difficult (2).

Pathogenic variants at the locus 13q12, DFNB1 (*GJB2* and *GJB6* genes), account for about half of all DFNB cases and are the most common cause of severe-to-profound DFNB (3). The gap junction beta-2 (*GJB2*) gene encodes the protein connexin 26, which is an integral part of gap junctions in the cochlea. Variants in the *GJB2* gene show a regional/ethnic distribution. In European and American white populations, the most common variant responsible for about 70% of DFNB1 is c.35delG. The carrier frequency of this variant is particularly high in the European Mediterranean countries (4,5).

Most AD loci cause postlingual progressive HL, while prelingual HL is caused by DFNA3 (*GJB2* and *GJB6*), DFNA8/12 (*TECTA*), and DFNA19 loci. Missense mutations in *TECTA* are a well-known genetic cause of mid-frequency or high-frequency AD HL in different populations (6,7).

In 30% of hereditary HL cases, HL may be one of the features of about 400 different syndromes. Sometimes the symptoms of these syndromes are very mild, nonspecific, or do not manifest at all at an early age; therefore, it is difficult to distinguish NSHL from the syndromic form of HL (8).

Next-generation sequencing (NGS) strategies, clinical exome sequencing (CES), and whole exome sequencing (WES) have significantly contributed to the diagnosis of genetically and clinically heterogeneous conditions, including hereditary HL. They have increased the diagnostic yield and contributed to the discovery of new genes associated with HL (9).

This study is an update on our previous study on *GJB2-*related NSHL in Croatia (10). The current study encompassed more than five times the number of participants from the previous study, and was enhanced by the inclusion of CES. The aim was to report the genetic causes of NSHL in a retrospective study of patients referred from several clinical centers and to determine the diagnostic yield using a combination of multiplex ligation-dependent probe amplification method (MLPA) and Sanger sequencing with the more contemporary CES technology.

## Methods

### Patients

In the period between March 2006 and October 2023, 352 unrelated patients with HL from Croatia were referred to our Department of Medical and Laboratory Genetics, Endocrinology and Diabetology, Children’s Hospital Zagreb for NSHL genetic testing. Clinical geneticists at University Children’s Hospital Zagreb, University Hospital Centre Split, University Hospital Centre Osijek, and University Hospital Centre Zagreb evaluated patients by obtaining family history, and performing physical examination and additional laboratory testing when appropriate. Forty-six unrelated patients with additional features and symptoms suggesting syndromic or acquired HL (such as infection, trauma, noise, ototoxic drugs, and premature birth), those with adult onset HL, unilateral HL, and those with insufficient medical data were excluded from the study. The final sample consisted of 306 patients (169 men [55.2%]) who were diagnosed with childhood familial or sporadic, mild to profound NSHL by a clinical geneticist. Severity of HL was classified as follows: <20 dB, normal hearing; 20–39 dB, mild HL; 40–69 dB, moderate HL; 70–94 dB, severe HL; and >95 dB, profound HL (mean of hearing at 0.5–1–2– 4 kHz). The ethnicity of the patients was not recorded because the vast majority were white Eastern Europeans and/or of Slavic origin. The median age of probands at the time of clinical assessment was 3.4 years (41 months, range 1 month-44 years). Informed consent was obtained from all patients or, in the case of minors, from their parents/legal guardians.

### MLPA analysis and Sanger sequencing of the GJB2 gene

MLPA analysis was performed by using SALSA MLPA kit P163, version B1, C1, and D1 GJB WFS1 (MRC Holland, Amsterdam, The Netherlands), according to the manufacturer’s recommendations (11). The latest version, D1, is designed to detect deletions and duplications of the *GJB2, GJB3, GJB6, WFS1*, and *POU3F4* genes. This probemix contains six probes detecting the wild type *GJB2* sequence (transcript NM_004004.6) of the c.313del14, c.235delC, c.167delT, c.101T>C, c.35delG, and IVS1 + 1G>A variants. FAM-labeled PCR products were separated by capillary electrophoresis on an ABI PRISM® 310 or SeqStudio Genetic Analyzer and subsequently analyzed by GeneMapper (both from Applied Biosystems, Thermo Fisher Scientific, Waltham, MA, USA) and Coffalyser.Net software (MRC Holland). A reduced signal of one of the mutation-specific probes can point toward the presence of this mutation or a (partial) deletion of *GJB2*. If that was the case, Sanger sequencing of the target sequences in exon 2 of *GJB2* was performed for confirmation. The exon 1 and flanking donor splice site of the *GJB2* gene was amplified with primers described earlier (10).

In all patients in whom MLPA analysis did not show copy number variation, Sanger sequencing of the complete coding region (exon 2) of the *GJB2* gene was performed. The coding region was amplified and sequenced by using primer sequences described elsewhere (12). Sequencing was carried out with a BigDye v1.1 or v3.1 sequencing kit (Applied Biosystems, Thermo Fisher Scientific), followed by capillary electrophoresis on an Applied Biosystems ABI PRISM® 310 or SeqStudio capillary sequencer.

### CES – library preparation, sequencing, and bioinformatics

CES was implemented in our laboratory diagnostic setting in 2018. From April 2019 to October 2023, CES was performed in 21 patients who tested negative for *GJB2* biallelic variants. The DNA library for CES analysis was generated with enrichment oligos by using Illumina DNA Prep with Enrichment (Illumina Inc., San Diego, CA, USA) focusing on the exons of 4813 disease-associated genes (TruSight One Panel, Illumina Inc.). Sequencing was performed on Illumina’s MiniSeq, MiSeq, and NextSeq platforms in 2 × 150 pair-end reads. Variants were considered for interpretation if a minimum median coverage was 70 × and if they were covered by at least 20 reads.

### Variant interpretation

ClinVar, HGMD, LOVD, and Hereditary Hearing Loss Homepage databases were used to search for variants associated with NSHL (13-15). The clinical significance of the variants was assessed with the use of pathogenicity and conservation scores from VarSome Clinical and Ensembl Variant Effect Predictor. All variants were classified according to the American College of Medical Genetics and Genomics/Association for Molecular Pathology (ACMG/AMP) guidelines (16). Only pathogenic, likely pathogenic variants, and variants of uncertain significance (VUS) were considered as possible causes of NSHL. Common benign variants were not presented in this report because they were not the subject of research.

Clinically relevant variants were filtered according to phenotypes listed in the Human Phenotype Ontology database (HPO): Sensorineural Hearing Impairment (HP:0000407) and Hearing Impairment (HP:0000365). If NSHL was accompanied by some other clinical features, the variants in genes associated with additional HPO terms were also surveyed (17).

## Results

### Spectrum of variants in the GJB2 gene

**Figure 1:**
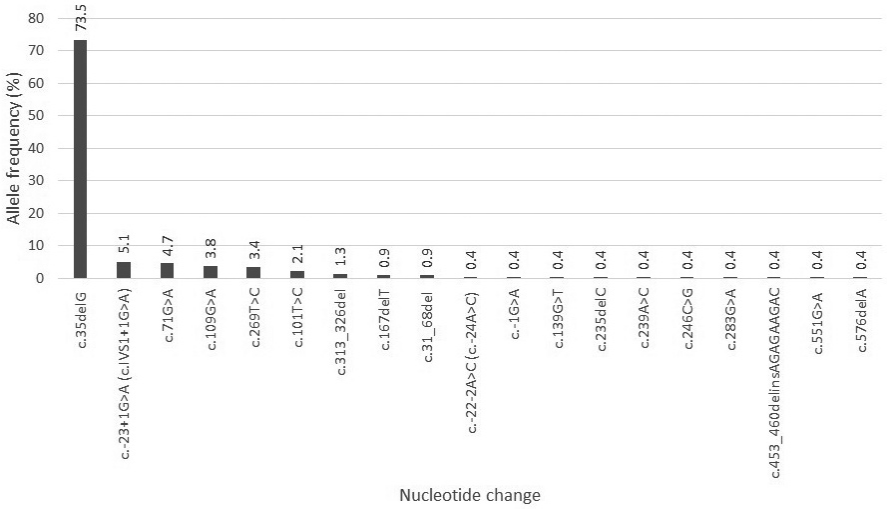
The spectrum and frequency of 234 disease-associated *GJB2* alleles detected in 306 unrelated patients with childhood-onset non-syndromic hearing loss.

Among 306 NSHL patients, 234 disease-associated *GJB2* alleles were detected. In total, 19 clinically relevant *GJB2* variants were identified, of which 18 were reported as pathogenic or likely pathogenic, and only c.-1G>A variant was categorized as a VUS. All *GJB2* variants detected have already been reported in individuals with NSHL (2). The most common variant by far was c.35delG, accounting for 73.5% of mutated alleles. It was followed by alleles c.-23 + 1G>A, c.71G>A, c.109G>A, c.269T>C, c.101T>C, and c.313_326del, whose frequency was 1.3%-5.1%. All other alleles were detected only once or twice. Except c. 551G>A, all alleles were associated with an AR mode of inheritance (Supplement 1(Supplementary material), Figure 1).

In 124 participants with *GJB2* variants, 110 had biallelic *GJB2* variants and 14 had only one *GJB2* variant. There were 20 different biallelic *GJB2* genotypes. More than half of the patients (64/110, 58.2%) with biallelic *GJB2* variants were 35delG homozygotes. The second most frequent genotype was c.[-23 + 1G>A];[35delG] compound heterozygotes. Only three types of *GJB2* alleles were represented in homozygotes: c.35delG, c.71G>A, and c.109G>A. The most common variant in compound heterozygotes was the c.35delG allele, which was present in 90.5% (38/42) of compound heterozygotes. The remaining 14 patients had a single heterozygous mutation. Again, in this group c.35delG was the most frequent allele, followed by c.109G>A and c.71G>A (Table 1).

**Table 1:** The genotypes of non-syndromic hearing loss (NSHL) patients with disease-causing biallelic and monoallelic *GJB2* variants. The reported variants are located in transcript NM_004004.6*

Genotype of patients with biallelic *GJB2* variants	No. (%) of patients with biallelic *GJB2* variants	
c.[35delG];[35delG], p.[Gly12fs];[Gly12fs]	64	(58.2)
c.[-23 + 1G>A];[35delG], p.[IVS1 + 1G>A];[Gly12fs]	11	(10.0)
c.[35delG];[269T>C], p.[Gly12fs];[Leu90Pro]	5	(4.5)
c.[35delG];[109G>A], p.[Gly12fs];[Val37Ile]	4	(3.6)
c.[35delG];[101T>C], p.[Gly12fs];[Met34Thr]	3	(2.7)
c.[35delG];[313_326del], p.[Gly12fs];[Lys105fs]	3	(2.7)
c.[35delG];[71G>A], p.[Gly12fs];[Trp24Ter]	3	(2.7)
c.[71G>A];[71G>A], p.[Trp24Ter][Trp24Ter]	3	(2.7)
c.[35delG];[c.31_68del], p.[Gly12fs];[Gly11fs]	2	(1.8)
c.[35delG];[167delT], p.[Gly12fs];[Leu56fs]	2	(1.8)
c.[109G>A];[109G>A], p.[Val37Ile];[Val37Ile]	1	(0.9)
c.[35delG];[c.139G>T], p.[Gly12fs];[Glu47Ter]	1	(0.9)
c.[35delG];[453_460delinsAGAGAAGAC], p.[Gly12fs];[p.Met151fs]	1	(0.9)
c.[-22-2A>C];[269T>C], p.[?];[Leu90Pro]	1	(0.9)
c.[35delG];[235delC], p.[Gly12fs];[Leu79fs]	1	(0.9)
c.[101T>C];[246C>G], p.[Met34Thr];[Ile82Met]	1	(0.9)
c.[109G>A];[576del], p.[Val37Ile];[Val193fs]	1	(0.9)
c.[35delG];[239A>C], p.[Gly12fs];[Gln80Pro]	1	(0.9)
c.[35delG];[283G>A], p.[Gly12fs];[Val95Met]	1	(0.9)
c.[-23 + 1G>A];[269T>C], p.[IVS1 + 1G>A];[Leu90Pro]	1	(0.9)
Total	110	
**Genotype of patients with one *GJB2* variant**	**No. (%) of patients with monoallelic *GJB2* variants**	
c.[35delG];[ = ], p.Gly12fs	6	(42.9)
c.[109G>A];[ = ], p.Val37Ile	2	(14.3)
c.[71G>A];[ = ], p.Trp24Ter	2	(14.3)
c.[551G>A];[ = ], p.Arg184Gln*	1	(7.1)
c.[269T>C];[ = ], p.Leu90Pro	1	(7.1)
p.[101T>C];[ = ], p.Met34Thr	1	(7.1)
c.[-1G>A];[ = ]^†^	1	(7.1)
Total	14	

*autosomal dominant variant.

†variant of uncertain significance.

### Spectrum of variants in non-*GJB2* genes

Seventeen non-*GJB2* variants were found in 10 genes (*TECTA, NOG, SLC26A4, PCDH15, TMPRSS3, USH2A, GATA3, MYO15A, SOX10, COL2A1*) in 11 participants. A single variant was found in four genes (*NOG, GATA3, SOX10,* and *COL2A1*), two variants were found in five genes (*SLC26A4, PCDH15, TMPRSS3, USH2A,* and *MYO15A*), and three variants were found in *TECTA* (Table 2). Sixteen variants were classified as pathogenic or likely pathogenic, and one variant was classified as of unknown significance (c.650C>T variant in *TECTA*). Two variants in *TECTA* and one in *NOG, PCDH15,* and *SOX10* were novel, accounting for 29.4% (5/17) of the total number of non-*GJB2* variants. In six of the 11 probands, a heterozygous variant in the *TECTA, NOG, GATA3, SOX10,* and *COL2A1* genes resulting in AD HL was detected. Compound heterozygous disease-causing variants were identified in five probands in genes associated with AR HL (*SLC26A4, PCDH15, TMPRSS3, USH2A*, and *MYO15A*). None of the variants were found in the homozygous state. One female patient had two pathogenic heterozygous variants: one associated with AD syndromic HL and the other associated with the non-HL phenotype. The studied group consisted of five familial (*MYO15A*, *NOG, SLC26A4*, and *TECTA* twice) and six sporadic cases of HL (*TECTA, GATA3, PCDH15, TMPRSS3, USH2A, and SOX10/ COL2A1*).

**Table 2:** Non-*GJB2* disease-causing variants in 11 patients with non-syndromic hearing loss (NSHL) and syndromic HL detected by clinical exome sequencing of 20 patients*

Patient ID	Gene/ transcript	Variant	Protein change	Variant type	Zygosity	Inheritance	Occurrence of HL in family	Phenotype	ClinVar Variation ID/citation	Pathogenic criteria	ACMG/AMP classification
**P4916**	TECTA/ NM_005422	c.6167G>T	*p.Cys2056Phe*	missense	Het	AD	familial	DFNA8/12	Novel variant	PP3, PP1, PM1, PM2	Likely Pathogenic
**P0914**		c.6183G>T	p.Arg2061Ser	missense	Het		familial		(18)	PP1, PP3, PP4, PM2	Likely Pathogenic
**P41920**		c.650C>T	*p.Thr217Ile*	missense	Het		sporadic		Novel variant	PM2, PM1	VUS
**P19613**	NOG/ NM_005450	c.291delC	*p.Ala98Argfs*	frameshift	Het	AD	familial	Teunissen-Cremers syndrome	Novel variant	PVS1, PM2, PP1, PP4	Pathogenic
**P01213**	SLC26A4/ NM_000441	c.1334T>G	p.Leu445Trp	missense	Het	AR	familial	Pendred syndrome/ DFNB4	ClinVar ID: 4829	PS3, PP2, PP3, PM2, PM3, PP4, PP5	Pathogenic
		c.918 + 1G>T	NA	splice site	Het				ClinVar ID: 43571	PVS1, PM2, PM3, PP3, PP4	Pathogenic
**P34720**	PCDH15/	c.794A>G	*p.Asp265Gly*	missense	Het	AR	sporadic	DFNB23/Usher syndrome, type 1F	Novel variant	PP3, PM3, PM2, PP4	Likely Pathogenic
	NM_001142763	c.2012 + 1G>A	NA	splice site	Het				ClinVar ID: 379720	PVS1, PP5, PM2, PP4	Pathogenic
**P57319**	TMPRSS3/ NM_024022	c.208del	p.His70Thrfs	frameshift	Het	AR	sporadic	DFNB8/10	ClinVar ID: 165492	PVS1, PP5, PM2	Pathogenic
		c.271C>T	p.Arg91Ter	nonsense	Het				ClinVar ID: 996721	PVS1, PP5, PM2	Pathogenic
**P44118**	USH2A/ NM_206933	c.4933G>T	p.Gly1645Ter	nonsense	Het	AR	sporadic	Usher syndrome, type 2A	ClinVar ID: 863680	PVS1, PP5, PM2, PM3	Pathogenic
		c.11140C>T	p.Gln3714Ter	nonsense	Het				(19)	PVS1, PM2, PM3	Pathogenic
**P47917**	GATA3/ NM_001002295	c.404dup	p.Ala136Glyfs	frameshift	Het	AD	sporadic	Barakat syndrome	ClinVar ID: 2136840	PVS1, PS2, PP5, PM2	Pathogenic
**P11616**	MYO15A/ NM_016239	c.8183G>A	p.Arg2728His	missense	Het	AR	familial	DFNB3	ClinVar ID: 228276	PM1, PM2, PM3, PP3, PP5	Pathogenic
		c.9518-2A>G	NA	splice site	Het				ClinVar ID: 2444220	PVS1, PM2, PP5, PM3	Pathogenic
**P03215**	SOX10/ NM_006941	c.456dup	*p.Phe153Leufs*	frameshift	Het	AD	sporadic	Waardenburg syndrome, type 4C	Novel variant	PVS1, PS2, PM2, PP4	Pathogenic
	COL2A1/NM_001844.4	c.2668C>T	p.Gln890Ter	nonsense	Het	AD	familial	Stickler syndrome, type I, nonsyndromic ocular	ClinVar ID: 2033750	PVS1, PP1, PP5, PM2	Pathogenic

*Abbreviations: NA – not applicable; Het – heterozygous; AD – autosomal dominant; AR – autosomal recessive; ACMG/AMP – American College of Medical Genetics and Genomics/Association for Molecular Pathology.

Acknowledgement We thank the families and patients for their cooperation.

Funding This study was supported by the Scientific Center of Excellence for Reproductive and Regenerative Medicine, Republic of Croatia, and by the European Union through the European Regional Development Fund, under grant agreement No. KK.01.1.1.01.0008, project “Reproductive and Regenerative Medicine – Exploring New Platforms and Potentials.”

Ethical approval granted by the Ethics Committee of the Children’s Hospital Zagreb University of Zagreb School of Medicine (02-23/13-2-18).

**Figure 2:**
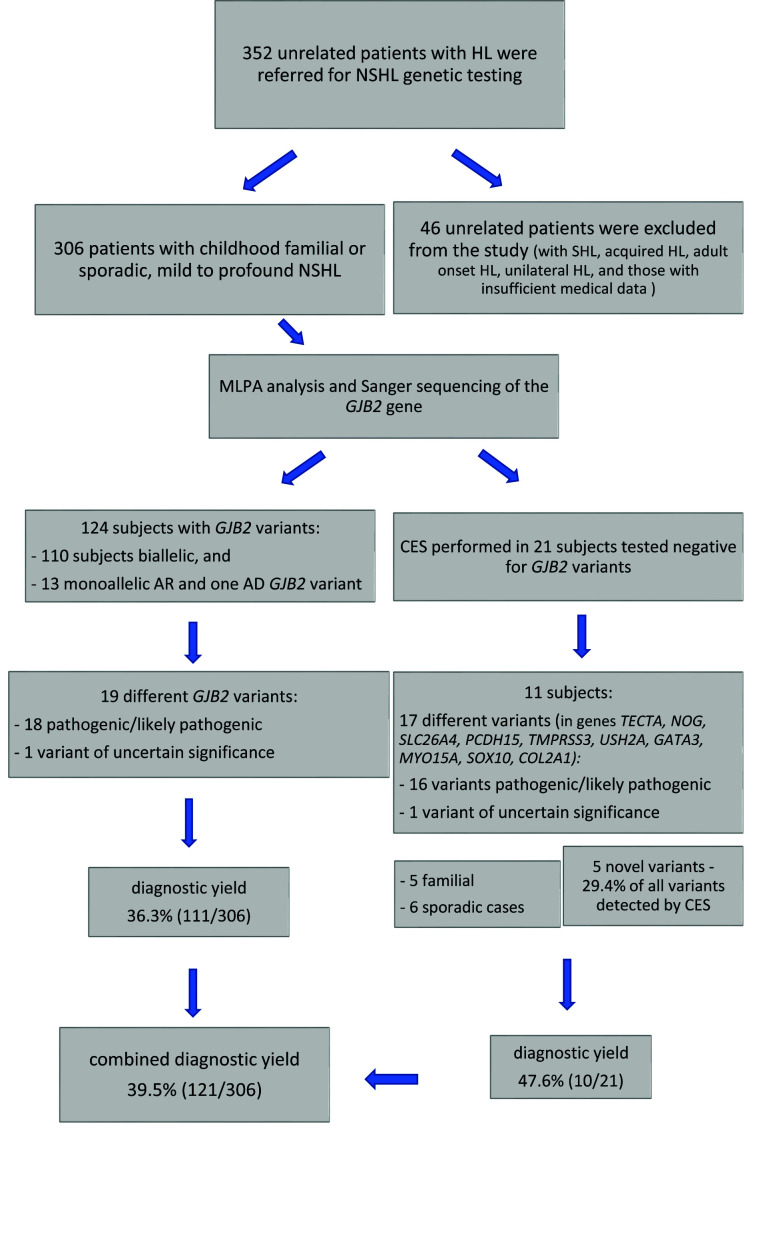
The flow diagram of genetic testing of patients with non-syndromic hearing loss. *Abbreviations: HL – hearing loss; NSHL – non-syndromic hearing loss; SHL – syndromic hearing loss; MLPA – multiplex ligation–dependent probe amplification; AR – autosomal recessive; AD – autosomal dominant; CES – clinical exome sequencing.

With the exclusion of 13 individuals with monoallelic *GJB2* recessive mutations, the diagnostic yield of the molecular testing of the *GJB2* variants by MLPA and Sanger sequencing in NSHL patients was 36.3% (111/306). By CES analysis performed on 21 participants, we were able to elucidate the genetic cause of HL in 10 patients (47.6%), without considering one VUS variant in the *TECTA* gene (Figure 2).

## DISCUSSION

Compared to the previous study of 58 NSHL patients from Croatia in which seven HL-related variations were found (c.35delG, c.71G>A, c.109G>A, c.269T>C, c.313_326del, c.23 + 1G>A, and c.-22-2A>C), in this study, 12 additional clinically relevant *GJB2* variants were detected (10) (Figure 1). As in our previous NSHL studies, the 35delG allele was the dominant pathogenic *GJB2* allele among individuals with NSHL (10,20). 35delG homozygotes were the most represented *GJB2* genotype, and the 35delG allele was present in almost all compound *GJB2* heterozygotes (90.5%). Such a high prevalence of the 35delG allele among *GJB2* genotypes is consistent with other NSHL studies of the white population in the USA and the European Mediterranean countries, which have a high frequency of 35delG carriers (4,5,12,21,22). The second most frequent genotype was c.[-23 + 1G>A];[35delG], detected in 11 patients. In Central European countries, c.[-23 + 1G>A];[35delG] genotype, as well as splice site variant c.-23 + 1G>A, is one of the most frequent pathogenic variants in the *GJB2* gene (23,24). In addition to the 35delG, variants c.71G>A, c.109G>A, c.269T>C, c.101T>C were found in a patient with biallelic, as well as in a patient with monoallelic *GJB2* variants. These variants were also detected at higher frequencies in other white populations (12,22-25).

Using CES, we found the genetic cause for syndromic HL in six probands, and one pathogenic variant causing non-HL phenotype in one patient. In two patients, we found disease-causing AR variants in the *USH2A* and *PCDH15* genes. Similar to other reported cases of Usher syndrome, USH2A and USH1F patients are often initially misdiagnosed with NSHL due to the absence of retinitis pigmentosa in early childhood (8).

In a 9.5-year-old girl with congenital severe bilateral HL, juvenile rheumatoid arthritis, and myopia, a *de novo* frameshift mutation c.404dup in the *GATA3* gene was identified. Heterozygous mutations in the *GATA3* gene are a known cause of Barakat syndrome (MIM#146255). However, the girl, except for HL and asymptomatic hyperthyroidism, did not show any other features of Barakat syndrome (26). Because of hyperthyroidism she was monitored by an endocrinologist. Her kidney function, which was normal at the time of examination, was monitored by a nephrologist.

In a 3.5-year-old girl with congenital moderate-to-severe progressive HL, we detected recessive biallelic pathogenic variants in the *SLC26A4* gene, which cause Pendred syndrome or DFNB4 disorder with an enlarged vestibular aqueduct (27). Brain magnetic resonance imaging (MRI) did not show enlarged vestibular aqueduct in the proband, but in her younger sister with the same type of HL, MRI showed bilaterally enlarged endolymphatic duct and sac. Probably, a computed tomography scan of the temporal bones, which was not performed in the proband, would show an enlarged vestibular aqueduct. At the time of the examination, her thyroid function was normal.

Five novel heterozygous variants accounted for 29.4% of all variants detected by CES analysis. There were two missense variants: c.650C>T and c.6167G>T in *TECTA;* one frameshift variant c.291delC in *NOG;* one missense variant c.794A>G in *PCDH15*, and one frameshift variant c.456dup in *SOX10* gene.

Participants tested by CES analysis most frequently had variants in *TECTA.* Variant c.650C>T categorized as a VUS was found in a premature baby boy with perceptive unilateral HL and mild specific learning and speech disabilities. The position was less conserved, and the *in silico* predictions were slightly more in favor of the benign nature of the variant, but this variant is extremely rare in the general population, and the proband`s parents had not yet been tested. Nevertheless, since clinical features of the boy were more suggestive of a non-genetic etiology of HL, we believe that the c.650C>T variant is probably benign.

A likely pathogenic c.6167G>T variant in *TECTA* was detected in a 9.5-year-old girl with moderate HL. The substitution replaced conserved amino acid cysteine at codon 2056 with phenylalanine in the zona pellucida (ZP) domain of α-tectorin. Missense variants in the ZP domain of α-tectorin predominantly cause mid-frequency HL. This variant was absent from the gnomAD exomes and gnomAD genomes database, and the multiple *in silico* predictions were in favor of the pathogenic nature of the variant. The proband`s multiple family members were affected wby HL: mother and father, both brothers, three siblings on the mother's side, and the mother's grandmother. Every family member was using hearing aids. Except for the father, all family members had prelingual HL. The audiograms of the proband and other family members with HL were not available. The mode of segregation of the variant in the family is unknown because the family did not undergo genetic testing.

In the *NOG* gene, the frameshift variant c.291delC was found in a two-year-old girl and her mother. The girl had facial dysmorphism, congenital moderately severe-to-severe bilateral progressive HL, convergent strabismus, amblyopia of the right eye, hypermetropia, short neck, accelerated bone growth, and partial syndactyly of the second and third toe on both feet. Her mother had conductive progressive HL, otosclerosis, hypermetropia, and slightly broader thumbs and big toes. The mother's father also had HL. The phenotype of the proband and her mother corresponds to the features of AD Teunissen-Cremers syndrome (MIM#184460) (28). The variant was categorized as pathogenic since it is a loss-of-function (LOF) variant that does not have a gnomAD exomes and gnomAD genomes entry, the phenotype of the family was highly specific to the above-mentioned syndrome, and there was segregation of the variant in the affected family members.

Two variants in the gene encoding protocadherin-15 (*PCDH15;* MIM*605514): pathogenic splice site donor variant c.2012 + 1G>A in intron 17 and novel likely pathogenic missense variant c.794A>G in exon 9, were found in a boy aged 3 years and 10 months with sensorineural and conductive, severe to profound bilateral HL. A homozygous or compound heterozygous mutation causes *PCDH15*AR deafness-23 (DFNB23; MIM#609533) and Usher syndrome type 1F (USH1F; MIM#602083). Patients carrying hypomorphic variants (some missense and in-frame with residual function) usually display profound neurosensory prelingual NSHL with normal vision (DFNB23), while those with more severe mutations (splice site, nonsense, frameshift) show USH1F phenotype characterized by severe to profound perceptive HL, vestibular dysfunction, and retinitis pigmentosa (29). However, missense mutations have also been described in patients with USH1F (30). The splice site variant has already been reported in homozygous and compound heterozygous individuals affected by USH1F (ClinVar ID:379720). According to the ACMG/AMP classification, the novel variant c.794A>G is likely pathogenic. The position is highly conserved, multiple *in silico* predictions favor the pathogenic nature of the variant, and it does not have gnomAD exomes/genomes entry. At the time of writing, the boy exhibited normal vision and vestibular function, and his parents had normal hearing. Since retinitis pigmentosa in people with USH1F has variable onset and can present itself later in adulthood, the patient was advised to have regular vision controls.

Two heterozygous autosomal dominant variants in the *SOX10* (MIM*602229) and *COL2A1* genes were detected in a girl aged 9.5 years with profound sensorineural bilateral HL, iris heterochromia, anosmia, cataract, and high myopia. Nobody in her family had HL, but her father had juvenile cataract. At the c.456 position in exon 3 of the *SOX10* gene, *de novo* frameshift indel variant was found (p.Phe153LeufsTer6). Pathogenic heterozygous mutations in this gene have been associated with autosomal dominant Waardenburg syndrome (WS) type 2E (WS2E; MIM#162200) and Waardenburg syndrome type 4C (WS4C; MIM #613266). According to the ACMG criteria, this variant is considered pathogenic. LOF is a known pathologic mechanism in this disease. The variant is very rare, and it has not been described in the gnomAD, ClinVar, HGMD, and LOVD databases, or in the available literature. The proband`s phenotype corresponded more to the WS4C features, since individuals with WS2E may have neurologic abnormalities. However, a variable phenotype and incomplete penetrance of some features have been reported in both WS disorders (31). The nonsense variant c.2668C>T (p.Gln890Ter) in the *COL2A1* (MIM*120140) gene is classified as pathogenic in one submission in ClinVar, but the phenotype of the tested individual is not known. It is not described in other databases or in the available literature. *COL2A1* pathogenic variants typically result in premature termination of translation and decreased synthesis of type II collagen. Changes in this gene are associated with various AD collagen disorders, including the non-syndromic, ocular form of Stickler syndrome, type I (MIM# 609508) (32). Since this *COL2A1* variant is inherited from the patient’s father with normal hearing, we assume that this variant is a cause of the ocular phenotype present in both affected family members. The non-syndromic form of Stickler syndrome type I is usually not associated with sensorineural HL. Therefore, a defect in *SOX10* alone is very likely responsible for the HL phenotype in the proband.

*TECTA*-related AD NSHL is one of the most frequent subtypes of AD NSHL (6). *TECTA* is comprised of 23 exons, and more than 80% of missense variants in *TECTA* submitted in the ClinVar database are of uncertain significance (13). Many of these variants are private, meaning that they occur in a very small number of cases, usually familial. Therefore, it is challenging to decipher missense *TECTA* variants causing AD NSHL. In this study, we detected two likely pathogenic *TECTA* variants in families with multiple members affected by moderate-to-severe NSHL. While determining the clinical significance of variants, one should use not only different *in silico* prediction tools, but also the phenotypes of patients and the study of family segregation (18).

The diagnostic rates of applied genetic methods were comparable with those reported previously (3,33). The reason for the slightly higher detection rate (44%) of pathogenic *GJB2* variants in the previous study is probably the sample size bias, that is, the higher representation of patients with congenital, AR severe-to-profound NSHL in a relatively small cohort compared with the current study. We must emphasize that CES analysis covered only 10.8% (21/195) of patients negative for *GJB2* biallelic variants. Nevertheless, it allowed us to elucidate the genetic cause of HL in 10 more patients, resulting in a total diagnostic yield of 39.5% in the NSHL cohort.

This study has some minor limitations. Some participants had insufficient audiological data (eg, audiograms, progression of HL), incomplete family history, and irregular follow-up examinations, due to which we were not able to show the *GJB2* genotype-phenotype correlation. It is difficult to recruit some HL-families with multiple members affected (most often both parents) for genetic testing because they do not consider HL a disorder but a special condition that should not be tested or treated as such. Fortunately, with today's cochlear implant options and those offered by gene therapy, more and more people are getting involved in genetic testing for HL. Regarding the methodology, 89.2% (174/195) of *GJB2*-negative patients were not analyzed by CES. The reasons for this were as follows: 1) the implementation of CES analysis started only in 2018, when some adult patients were no longer followed up in pediatric clinics and 2) the small capacity of MiSeq and MiniSeq genomic analyzers, which were used most of the time in CES. This study did not include copy number variant (CNV) analysis in NSHL genes due to the insufficient number of patients analyzed. Since recent studies have shown that CNVs contributed to the genetic diagnosis of NSHL in 15.2% of patients, CNV analysis, especially of the *STRC* and *OTOA* genes, remains to be implemented in further studies (34,35).

In conclusion, all *GJB2* variants detected in this study have already been reported in individuals with NSHL, but almost one third of all variants detected by CES were novel. The c.35delG was the most common variant. In the non-*GJB2* gene group, the most variants were found in *TECTA*. With applied methods, we were able to elucidate the genetic cause of HL in 39.5% of patients. CES enabled us to diagnose almost half of the patients with HL (10/21); distinguish NSHL from the syndromic form of HL in cases where the phenotype was indistinctive or symptoms were absent at an early age, such as in retinitis pigmentosa in Usher syndrome; and discover an additional genetic cause of non-HL phenotype in a patient with syndromic HL (WS4C). Nevertheless, considering the high prevalence of *GJB2* variants among the tested NSHL Croatian participants, the recommended NSHL testing strategy would still be screening for the *GJB2* variants with MLPA and Sanger sequencing, followed by NGS (CES or WES) analysis in patients negative for *GJB2*-biallelic variants.
