# Supplementary material for: Spectrum of genetic variants in 306 patients with non-syndromic hearing loss from Croatia
Source: Croat Med J. 2024 Jun;65(3):198–209. doi: 10.3325/cmj.2024.65.198 (PMC11157262; doi:10.3325/cmj.2024.65.198)
Supplement: Supplementary Material [file CroatMedJ_65_s006.pdf]

Supplemental Table 1. The spectrum and frequency of 234 disease-causing *GJB2* variants detected in 306 unrelated patients with childhood NSHL*

**Nucleotide change (NM_004004.6)**	**Protein change**	**Variant type**	**Effect**	**No. of alleles**	**Allele frequency (%)**
c.35delG	p.Gly12fs	Deletion/Frameshift	Pathogenic	172	73.5
c.-23+1G>A (c.IVS1+1G>A)	No mRNA	Splice site	Pathogenic	12	5.1
c.71G>A	p.Trp24Ter	Nonsense	Pathogenic	11	4.7
c.109G>A	p.Val37Ile	Missense	Pathogenic	9	3.8
c.269T>C	p.Leu90Pro	Missense	Pathogenic	8	3.4
c.101T>C	p.Met34Thr	Missense	Hypomorphic recessive allele	5	2.1
c.313_326del	p.Lys105fs	Deletion/frameshift	Pathogenic	3	1.3
c.167delT	p.Leu56fs	Deletion/frameshift	Pathogenic	2	0.9
c.31_68del	p.Gly11fs	Deletion/frameshift	Pathogenic	2	0.9
c.-22-2A>C (c.-24A>C)	residual expression of wild-type protein	Splice site	Likely pathogenic	1	0.4
c.-1G>A	Unknown	Splice site	VUS	1	0,4
c.139G>T	p.Glu47Ter	Nonsense	Pathogenic	1	0.4
c.235delC	p.Leu79fs	Deletion/Frameshift	Pathogenic	1	0.4
c.239A>C	p.Gln80Pro	Missense	Pathogenic	1	0.4
c.246C>G	p.Ile82Met	Missense	Pathogenic	1	0.4
c.283G>A	p.Val95Met	Missense	Pathogenic	1	0.4
c.453_460delinsAGAGAAGAC	p.Met151fs	Indel/Frameshift	Pathogenic	1	0.4
c.551G>A	p.Arg184Gln	Missense	Pathogenic	1	0.4
c.576delA	p. Val193fs	Deletion/Frameshift	Pathogenic	1	0.4
			Total mutated alleles (∑N)	234	

***Abbreviations:** NSHL - non-syndromic hearing loss; VUS-variant of uncertain significance
